# Long-Term Outcomes After Multidisciplinary Treatment for Pediatric Orbital Rhabdomyosarcoma

**DOI:** 10.3390/cancers17040615

**Published:** 2025-02-11

**Authors:** Nur Khatib, Johannes H. M. Merks, Jeroen E. Markenstein, Brian V. Balgobind, Cemile. D. Savci-Heijink, Michele Morfouace, Bradley R. Pieters, Peerooz Saeed

**Affiliations:** 1 Orbital Center, Department of Ophthalmology, Amsterdam University Medical Centers, 1105 AZ Amsterdam, The Netherlands; p.saeed@amsterdamumc.nl; 2 Princess Maxima Center for Pediatric Oncology, 3584 CS Utrecht, The Netherlands; 3 Department of Radiology, Amsterdam University Medical Centers, 1105 AZ Amsterdam, The Netherlands; j.e.markenstein@amsterdamumc.nl; 4 Department of Radiation Oncology, Amsterdam University Medical Centers, 1105 AZ Amsterdam, The Netherlands; b.v.balgobind@amsterdamumc.nl (B.V.B.); b.r.pieters@amsterdamumc.nl (B.R.P.); 5 Department of Pathology, Amsterdam University Medical Centers, 1105 AZ Amsterdam, The Netherlands; cemiles.h@amsterdamumc.nl

**Keywords:** rhabdomyosarcoma, pediatric rhabdomyosarcoma, orbital tumors, AMORE (mold after loading brachytherapy and surgical reconstruction)

## Abstract

(1) Background: Orbital rhabdomyosarcoma is a rare and aggressive soft tissue tumor that primarily occurs in the eye socket (orbit) of children. Treatment usually involves a combination of surgery, chemotherapy, and radiation therapy, aiming to remove the tumor and prevent metastasis. (2) Methods: An institutional retrospective study was conducted with data from 39 patients with primary orbital RMS treated between 1995 and 2016 at the Amsterdam University Medical Centers/Emma Children Hospital. (3) Results: The median age at presentation was 7 years (range, 9 months to 16 years). The median follow-up period was 9.4 years (range, 3 to 25 years). Ten underwent chemotherapy and excision without additional radiotherapy. A total of 29 patients received additional local treatment: Ablative surgery MOld technique with after loading brachytherapy and surgical REconstruction (AMORE) (N = 21), proton (N = 4) or external beam radiation treatment (EBRT; N = 4). We found 14 cases with recurrences, 9 of which underwent exenteration and two of which died. The 10-year overall survival rate was 95% and the EFS was 63%. (4) Conclusions: long-term follow-up with 10-year survival rate of orbital RMS in this series was 95% achieved by local tumor control and eye preservation in 77% of our study population.

## Simple Summary

Research on orbital rhabdomyosarcoma in children is vital for several reasons. Given the rarity and aggressive nature of this cancer, understanding its biology and behavior is crucial for developing effective treatment strategies. Additionally, studies focused on treatment modalities can lead to advancements in chemotherapy regimens and radiation techniques, ultimately aiming to enhance survival rates while minimizing long-term side effects. This study emphasizes the importance of achieving local tumor control with preservation of the eye form and function among our treated patients with high (95%) survival rate.

## 1. Introduction

Soft tissue sarcomas are a heterogeneous group of rare mesenchymal tissue malignant neoplasms that account for 7% of malignant neoplasms in children. Rhabdomyosarcoma (RMS) constitutes 50% of soft tissue sarcomas in children, and 10% of RMS cases occur in the orbit at a median age of 6.8 years [1,2].

Orbital RMS often presents mild symptoms. However, more severe or longer duration of symptoms should alert the physician to consider the presence of RMS, as this requires a swift and accurate diagnostic approach. Therefore, physicians should be aware of this tumor, recognize its clinical features, and refer patients for prompt diagnosis and treatment.

The orbit is a known favorable localization for RMS, with good prognosis. Following the efforts of European and North American collaborative groups, dramatic advances have been made in the understanding of the pathogenesis and management of RMS. As a result, the survival rate following treatment of RMS at all sites has improved from 25% in the early 1970s to 80% after the establishment of treatment protocols that include surgery, chemotherapy, and irradiation. In general, the current treatment approach starts with an incisional biopsy and staging, followed by neo-adjuvant chemotherapy and local therapy, often radiotherapy, after the first four chemotherapy cycles [2,3,4].

In the 1990s, a new treatment protocol termed Ablative surgery MOld technique with after loading brachytherapy and surgical REconstruction (AMORE) was developed [5,6,7,8]. The steep dose gradient of brachytherapy enables high dose delivery to the areas where microscopically tumor-free margins could not be obtained, whereas the surrounding healthy tissue receives an individually based relatively low radiation dose. This is aimed at reducing radiation damage to important structures in the orbit and surrounding tissues and organs. This could potentially induce fewer adverse events when compared to external beam radiation treatment (EBRT) [9,10]. However, AMORE does include surgery which might introduce other risks. Besides that, AMORE has specific feasibility criteria related to surgical resection which do not apply to external radiation with photons or protons. Essentially, there are now very effective conservative treatment options for many orbital tumors. In some cases, a surgical procedure can be avoided, and good visual function can be retained [11].

The aim of our study was to evaluate the treatments and outcomes of 39 consecutive cases of RMS affecting the orbit treated at our center using different treatment approaches over a period of 20 years.

## 2. Materials and Methods

### 2.1. Eligibility Criteria

This retrospective study was conducted at the Orbital Center of the Amsterdam University Medical Centers and included children aged 0–18 years diagnosed with and treated for primary RMS of the orbit between 1995 and 2016. The thirty-nine consecutive patients were from our center and had been treated according to our institution’s policy.

We reviewed all medical records of cases with histologically proven orbital RMS for demographic information (age, sex, and ethnic origin), clinical data history (including symptoms at presentation), imaging studies (reviewed by the same radiologist [author JM]), histopathologic information (reviewed by [author CS]), treatment received (all 39 cases were reviewed by a multidisciplinary panel), and events during follow-up.

### 2.2. Diagnostic and Follow-Up Procedures

#### 2.2.1. Imaging

We collected magnetic resonance imaging (MRI) and computer tomography (CT) images from the time of diagnosis, before local therapy, at end of treatment, at recurrence where applicable, and from the last follow-up. As part of regular follow-up, patients underwent orbital imaging every three months in the first year of follow-up, every 4 months in the second and third year of follow-up and yearly thereafter till five years follow up.

We categorized regions of the orbit as follows: superior, supratemporal, temporal, infratemporal, inferior, infranasal, nasal, and supranasal in sagittal sections. Tumors with bony erosion or invading parameningeal sites were considered as parameningeal tumors. Parameningeal sites are defined as sites adjacent to the meninges (nasopharynx, middle ear, paranasal sinuses, infratemporal and pterygopalatine fossa).

#### 2.2.2. Pathology

We reviewed all histopathological and immunohistochemistry (IHC) data on immune reactivity for myogenin, desmin, and vimentin, assessing their expression rates and positivity on the RMS diagnoses. Open surgical biopsies were revised and classified according to the International Society of Pediatric Oncology (SIOP) pathology guidelines [12].

#### 2.2.3. Staging

Tumors were classified according to the clinical tumor, node, metastasis (TNM edition 7,8) system of the Union for International Cancer Control (UICC) and the Intergroup Rhabdomyosarcoma Study (IRS) postsurgical staging system [13,14]. The IRS system classification is based on clinical aspects including local invasion, the post-initial-surgery status, and the presence of regional or distant metastases. The system classification has four groups: group I—microscopic complete resection, localized disease (excisional biopsy); group II—microscopic disease remaining after excisional biopsy; group III—gross residual disease remaining after incisional or true cut biopsy; group IV—distant metastasis present at onset (see Table 1). For the TNM classification, tumor localizations and extensions were assessed with MRIs and CT scans. Lymph node status was based on clinical examination, radiographic studies, and/or biopsy results. The workup for distant metastasis included chest imaging (Chest X-ray till until 2005, Chest CT from 2006 onwards), skeletal scintigraphy (from 2006 increasingly replaced by fluorodeoxyglucose-positron emission tomography/CT), cerebrospinal fluid analysis (only in case of parameningeal extension), and bone marrow aspirates and trephines.

Site definitions were in accordance with the SIOP malignant mesenchymal tumor (MMT) and European pediatric soft tissue sarcoma Study Group (EpSSG) guidelines. Subsequently, patients were stratified into risk groups based on age, tumor site, size, IRS post-surgical stage, nodal status, and histology. Chemotherapeutic treatment was based on this risk-group stratification, following the prevailing protocols [15,16].

### 2.3. Evaluation of Risk Factors

We assessed tumor status, parameningeal site involvement, age at presentation, and tumor size at diagnosis (</> 5 cm) as part of risk factors’ evaluation.

### 2.4. Outcome Assessment

Follow-up was performed every three months for the first year as standard of care. Follow-up included full ophthalmological examinations, orthoptic evaluation, routine slit lamp examination, optical coherence tomography, and fluorescein angiography (in patients with suspicion of retinopathy). Best-corrected visual acuity (BCVA) was evaluated using age-adapted tests: in children older than six years we used the Snellen BCVA chart, in children younger than 6 years we used the E cube test for cooperative children, and we used CSM (Central, Steady, Maintenance) evaluations (CSM, 0.6–1; CSNM, 0.16–0.1; unsteady central fixation < 0.1) for non-cooperative children. To differentiate between tumor and treatment effects on the visual acuity, BCVA outcomes before and after treatment were collected and compared. The definition of possible retinopathy involved the presence of at least one hemorrhage or microaneurysm on a standardized 508 red-free, achromatic retina photograph. We used 508 non-stereo, red-free photography, a technique slightly modified from that described and evaluated by the EURODIAB insulin-dependent diabetes mellitus complications study. For evaluation of dry eye, slit lamp examination was conducted with fluorescein. Then, all patients underwent a Schirmer test with signs of corneal dryness classified as: absence of corneal changes, corneal stippling limited to the inferior periphery, extended stippling, ulceration, and clouding.

Cataract was defined according to the Lens Opacity Classification System II (LOCS II) criteria which includes four classes: nuclear color, nuclear opalescence, cortical grading, and posterior subcapsular (PSC). Cataract was confirmed by a rating >1 for any of the classes and/or a rating ≥1 for PSC. Patients who had undergone cataract surgery were classified as having had cataract, and the LOCS II system was not used.

### 2.5. Treatment

From 1995 to 2005 all patients were treated according to the SIOP-MMT95 protocol and patients treated from 2005 onward were treated according to the EpSSG RMS2005 protocol. In low-risk patients achieving complete remission (CR) after initial (diagnostic) surgery and induction chemotherapy (2 or 3 courses), radiotherapy could be withheld. Patients with residual disease after initial (diagnostic) surgery and induction chemotherapy were evaluated by a multidisciplinary team (orbital surgeon, radiation oncologist, reconstructive surgeon, radiologist, and pediatric oncologist) for feasibility of a macroscopic radical resection followed by brachytherapy. Patients were considered eligible for this combined approach (AMORE) when infiltration of the orbital apex, extraocular muscles, and bulbar involvement were absent and macroscopic complete resection while maintaining ocular function was therefore feasible.

In patients eligible for AMORE, the tumor was macroscopically resected and an individually constructed mold (formed from 5 to 7 mm-thick, soft silicon blocks) was placed into the tumor bed. Flexible catheters loaded with iridium-192 were placed and fixed into tumor bed. By the end of this procedure X-ray or CT was performed to insure the catheter correct position. A dose of 40 Gy to 50 Gy was delivered to the wound bed, depending on risk factors and patient age. Furthermore, the space in the orbit is often limited and in cases with suboptimal mold placement, a larger heterogeneous dose distribution was acceptable to achieve an effective dose to the complete target volume. Affected bone was resected. When AMORE was not deemed feasible, alternative local treatment options included EBRT with photons or protons. In this case, the entire orbit received a dose of 36 Gy, after which a further dose of 41–50.4 Gy was delivered based on the initial tumor volume [17,18,19].

### 2.6. Statistical Analysis

Differences between two or more categorical variables were assessed using the chi-squared test. Median values between two groups were compared using the Mann–Whitney U test and for comparisons between >2 groups, analysis of variance (ANOVA) was used. We analyzed trends across categories using Cuzick’s non-parametric test for trends.

The Kaplan–Meier survival curve was used to calculate event-free survival (EFS) and overall survival (OS). Survival data were analyzed using SPSS version 20.0 (SPSS, Chicago, IL, USA). We calculated OS from diagnosis to the date of death from any cause and EFS from primary treatment point to the date of the first event, defined as progressive disease, relapse, or death from any cause. The cut-off point of the analysis was the 1st day of April 2021 and data from patients without an event were excluded after this date.

We evaluated the presence of the following potential prognostic risk factors for RMS: age at presentation, histology, primary tumor site (in orbital tumors), size (<5 cm/≥5 cm), stage, IRS post-surgical stage, extent (nodal/distant metastasis), erosion of bony boundaries, bone involvement, chemotherapy regimen, surgical approach, radiotherapy, and response to therapy.

We defined statistical significance as *p* < 0.05, and calculated this using the SPSS software for Windows, version 10 (SPSS, Chicago, IL, USA).

## 3. Results

Thirty-nine patients were included in this study. The median age at presentation was 7 years (range, 9 months to 16 years). Overall, 28 boys (72%) and 11 (28%) girls were included. Eyelid swelling was the most common presenting clinical manifestation in 19 patients (49%), followed by proptosis in 12 (31%), medial canthal swelling in 4 (10%), double vision in 3 (8%), and pain in 2 patients. Symptoms were localized to the right side in 22 patients (56%) and the left side in 17 patients (44%). Inflammation and orbital cellulitis were the most common primary diagnoses by referring physicians; these diagnoses mimicked RMS in 45% of the cases.

The median follow-up time in this series was 9.4 years (range, 3 to 25 years).

Visual acuity data were available from 29 patients who were cooperative during the examination. The mean BCVA of the ipsilateral eye among these 29 patients was 0.74 (median, 0.5; range, 0.25 to 1) at presentation. Among patients who did not undergo exenteration, the mean BCVA of the ipsilateral eye at the final follow-up was 0.84. Of the 29 patients; 62% had ipsilateral eye BCVA of 0.5 or better at the final follow-up visit and all had contralateral eye BCVA of 0.8 or better. Nine patients were blind in the ipsilateral eye after treatment due to exenteration, and one due to radiation optic nerve neuropathy.

### 3.1. Imaging

All 39 patients underwent CT or MRI scans of the primary tumor at diagnosis, depending on the hospital standard of care at that time. For 38 patients, images at both diagnosis and follow-up were available (and one loss of information), consisting of MRI data for 23 patients (59%) and CT data for 15 patients (38%).

**Figure 1:**
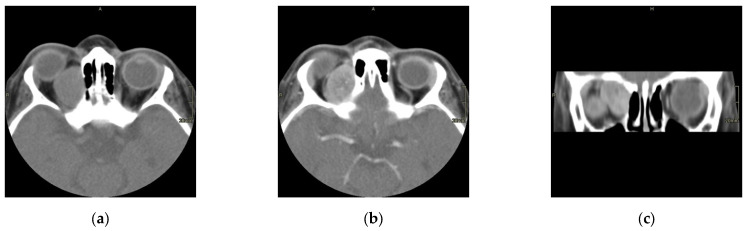
Computed scan tomography (CT) of orbital RMS case: non contrast axial section (**a**), contrast axial section (**b**), and contrast coronal section (**c**). This case shows a hypodense lesion on CT with post-contrast enhancement.

**Figure 2:**
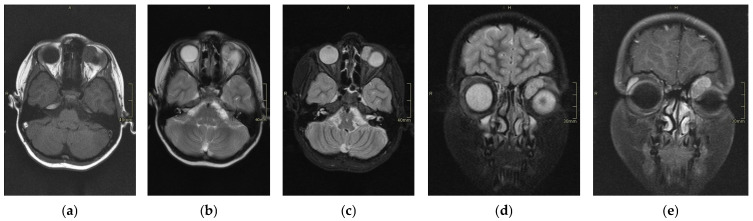
Magnetic resonance imaging (MRI) of RMS case: T1 axial section (**a**), T2 (normal) axial section (**b**), T2 with fat saturation axial section (**c**), T2 fat saturation coronal section (**d**), and T1 post-gadolinium (**e**). This case represents hypothesis T1, hyperintense T2, and intense enhancement postcontrast (gadolinium).

**Figure 3:**
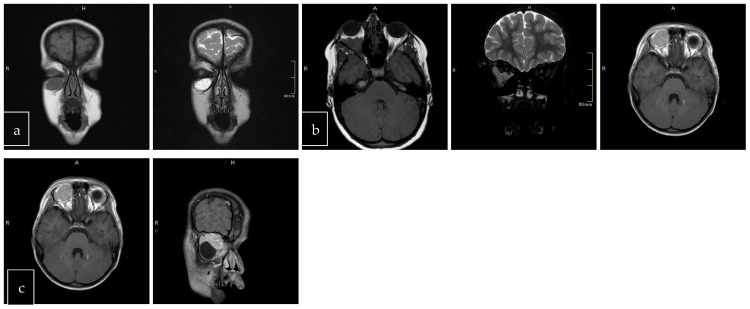
Tumor border featuring (smooth, lobulated, mixed); T1 and T2 coronal sections of a smooth (**a**). T1 axial and T2 coronal sections of lobulated mass (**b**). T1 axial post-contrast sections of mixed border lesion with both smooth (axial) and lobulated (coronal) (**c**).

The scans at diagnosis showed masses with hypodense contrast enhancement of the tumor in all cases, with a pattern of generalized enhancement (see Figure 1). MRI at diagnosis showed low signal (isointense or hypointense) of soft tissues on T1-weighted images and high signal (hyperintense) on T2-weighted images, and gadolinium enhancement in all cases (see Figure 2). The tumor border was smooth (in 77% of cases), lobulated (10%), or mixed (13%) (see Figure 3). No association was found between tumor border type and recurrence rate or death.

In 31 and 24 patients, respectively, information on maximum tumor diameter and tumor volume was available. The mean maximum tumor diameter at time of diagnosis was 2.9 cm (range, 0.9 cm to 7.4 cm) among the non-recurrence cases, and 2.5 cm (range 1.12 cm to 3 cm) among recurrence cases. The mean tumor volume at the time of diagnosis was 15.4 mL (50% of orbital volume). The mean maximum tumor diameter at time of recurrence was 2.8 cm (median 2.6; range, 1.7 cm to 3.9 cm) and the mean tumor volume was 12.8 mL.

More tumors were found in the medial than lateral half of the orbit (69% vs. 38.5%) and 26 of 38 tumors (68%) were located in the superior medial quadrant. All cases were extraconal, and in 18 of 38 (47%) patients we also found an intraconal invasion 10, of which (55%) showed apical invasion. We found rectus muscle involvement in 14 of 38 cases (37%). We found no perineural involvement in our study series. Pre-septal soft tissue involvement was observed in 26 of 38 patients (68%), four with lacrimal gland invasion. Parameningeal involvement was present in eight cases: five with lamina papyracea and sphenoid bone extensions, two with orbital roof involvement (one of which had intracranial invasion), and one with pterygopalatine fossa extension. Five cases were extensively multifocal orbital primary tumors, including extra/intraconal, muscles, lacrimal gland, apex, and bone.

Post-therapeutic contrast enhancement of the residual lesion was seen on follow-up imaging among 34 patients (89%). We diagnosed recurrences in 14 of these 34 patients (41% of postsurgical enhancement) during follow-up by image enhancement enlargement and final diagnostic biopsy.

### 3.2. Histopathology, IRS Classification, and Staging

Thirty-seven cases (95%) were of embryonal type and two (5%) were alveolar. Immunohistochemical (IHC) analysis revealed immune reactivity for desmin in all 36 tested cases (three cases with no information). Immuno-reactivity for myoglobin was detected in 24 among the 26 (92%) patients who underwent the test. Immune reactivity for vimentin was detected in nine of the 10 (90%) patients. FOXO1 fusion was positive in the one alveolar case for which IHC and fusion status were performed. Embryonal cases were not tested for FOXO1 fusion. According to the IRS post-surgical grouping, two cases were classified as group I, 11 cases as group II, and 21 cases as group III (Table 1). According to the TNM staging, 35 cases were T1N0M0, 3 cases were T2N0M0, and 1 case was T1N0M1 (Table 2).

### 3.3. Treatment Approach and Adverse Effects

The majority of patients (n = 37/39) underwent incisional biopsy during the primary diagnostic workup, and two patients (IRS group I) underwent primary excisional biopsy. All received chemotherapy according to the prevailing protocols at the time. (12, 13) Of 39 patients, 10 underwent chemotherapy and then excision without local radiotherapy. Of the other 29 patients, local treatment consisted of the AMORE protocol in 21, proton beam radiotherapy in 4, and external beam radiotherapy with photons in 4 as part of either primary or secondary treatment (see Table 3 cases; 3, 6, 20, 31, 42, 10). Of the 14 cases with recurrence, 9 (64%) underwent exenteration as part of their final treatment approach. Of the whole cohort, orbit and eye were preserved in 77% of patients. Retrospectively, muscle involvement was found in 70% of exenterated cases. Bone resections were performed in six out of seven cases with bone involvement, with one case undergoing exenteration.

Cataract was the most common late adverse effect of treatment in our study series, with 10 out of the 30 non-exenterated patients (33%) diagnosed as having cataract, and three of these needing cataract surgery. Six (out of 21) cases developed cataract after AMORE treatment, one (out of four) case after EBRT, one (out of four) case after proton beam treatment, and two (out of 10) cases were found spontaneously in non-locally treated patients as congenital cataract. Among the cases with recurrence, one of the five (20%) non-exenterated cases developed a cataract during the follow up period. The mean period between the patient’s first presentation and cataract diagnosis was 7.6 years (range, 3 to 20 years).

Out of the 29 cases (7%) that received radiation as part of their local treatment, 2 had radiation retinopathy. One case (no. 40 in Table 3) developed retinopathy as a result of secondary brachytherapy and was treated by laser with a final BCVA of 0.9. In the second case, retinopathy developed as a result of the primary AMORE procedure and was treated by anti-VEGF injections and laser, with final BCVA of 0.1. One of the twenty-nine patients developed optic disk atrophy following AMORE treatment, and this patient presented a unilateral painless blind phthisic eye at the final follow-up. Ptosis was observed in five patients (17%).

After the AMORE procedure, two patients experienced mild to moderate dry eye, which was locally treated. Three patients had enophthalmos and bone hypoplasia after the AMORE treatment approach. Two cases had impaired eye movement.

### 3.4. Recurrence and Survival

In total, we diagnosed 14 local recurrences in our single center cohort. All recurrences were local (no regional lymph node metastasis or distant metastasis), except one case with lung metastasis at the time of diagnosis (Table 4). All recurrent cases showed intraconal extension as the primary location. The recurrence site in all cases coincided with the primary location, except for one case which presented with recurrence in multiple locations of the orbit. All cases with recurrence underwent reevaluation by a multidisciplinary AMORE team. Our study reported two death cases, both diagnosed as embryonal histopathology; the cause of death in one case was intracranial spread and the other was loss of information.

Out of 14 recurrence cases, 10 were managed using AMORE as treatment approach, as were 8 out of 9 of the secondary recurrence cases (Table 3). We did not find a correlation between recurrent cases and histological morphology; all recurrent cases were diagnosed with embryonal histological morphology except for one which was diagnosed as alveolar.

The mean time from primary treatment to local recurrence was 36 months (median, 24; range, 7 to 120 months). Local recurrence was detected in six patients at the first-year follow-up, one patient at the second year of follow-up, one patient at the fifth year of follow-up, and two with exceptionally late recurrences nine years after initial treatment. However, in the latter cases, complete remission was achieved after chemotherapy, exenteration, and brachytherapy.

The frequencies of apical invasion and pre-septal involvement were high among the cases with recurrence; 8 of 14 (57%) showed apical extension, 10 of 14 (71.4%) showed extraconal involvement with pre-septal invasion, and 4 of 14 (28.6%) had lacrimal gland infiltration. Out of 5 cases with extensive orbital involvement (described in the Section 3.1), 3 had episodes of recurrence.

**Figure 4:**
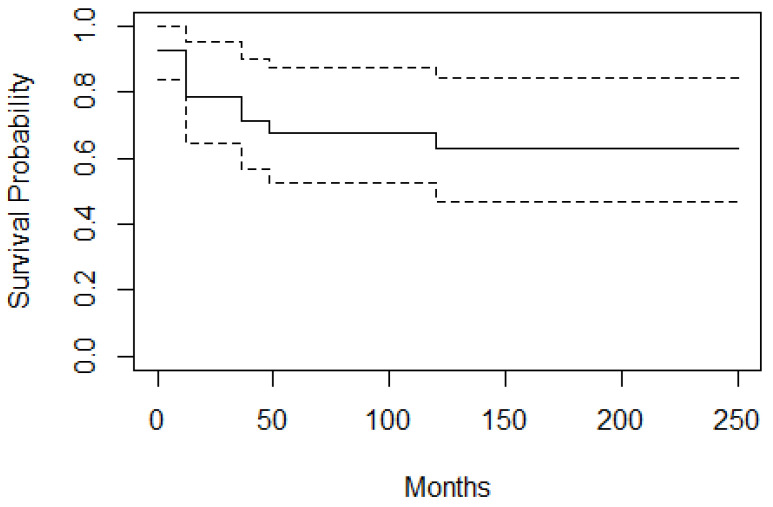
Event free survival curve; EFS is 63%. According to the curve at zero point, the chance of not experiencing the event is 92.3% because two cases had already shown treatment failure immediately after the primary treatment. At 12 months after starting the study, the chance of not experiencing the event is 78.6%.

Overall survival (OS) and EFS were calculated in 39 patients. The 10-year OS rate in our series was 95%. The survival rate between 9 months to 10 years was 96% and that of cases at older ages (≥10 years) was 93%. EFS (10 years) was found to be 63% [Figure 4].

### 3.5. Evaluation of Risk Factors

We found no statistically significant differences in these factors between the patients with or without recurrence.

## 4. Discussion

In the Netherlands, the most common pediatric malignant orbital tumor after lymphoma is RMS, with a relative incidence of 12% (44 RMS cases out of 367 orbital tumors were registered between 1989 and 2006) [18]. Our center has been the main national referral center for orbital RMS. The availability of AMORE treatment as a local treatment alternative for these patients is universally unique to our center. The choice for a local treatment strategy for each newly diagnosed patient involves a multidisciplinary team including an orbital surgeon, radiation oncologist, reconstructive surgeon, radiologist, and pediatric oncologist. Therefore, previous studies have been published over the last few years by our medical center which showed that the overall outcome for orbital RMS patients was good, and surgery and brachytherapy as a treatment modality for orbital RMS resulted in an effective local treatment approach with fewer adverse events than external beam radiotherapy [20]. To the previous literature, this present study presented the addition of a larger study group, a longer follow period, and the inclusion of identify factors predisposing treatment failure.

In the present study, the mean recurrence time was three years after diagnosis, which is similar to previous reports. All recurrences were local, with no systemic progression detected after primary treatment. The highest rates of recurrence occurred during the first year of follow-up, a critical period during which follow-up should take place at 3-monthly intervals after completion of therapy. The majority of our patients had local areas of post-treatment enhancement at the previous tumor site. These patients had MRIs every three months as part of their follow-up. During the follow up period, only 29% of these enhanced areas were diagnosed as recurrences after histological confirmation. According to Bita Esmaeli et al., a residual orbital mass on imaging may be present after multimodality treatment in approximately one-third of patients. Resolution without biopsy or excision varied from months to years [21]. Some authors have recommended that biopsies be obtained for suspected enhanced areas like these because persisting soft tissue abnormalities at follow-up are a prognostic indicator of a higher rate of relapse (the so-called “post-therapeutic residue”). However, we only advise biopsy when this residual enhancement is progressive, and careful observation at follow-up in non-progressive cases to avoid unnecessary procedures [19,22,23].

Our study lacked an exploration of the association between tumor diameter/volume and recurrence rate. Previous publications have shown that small (<5 cm) tumor size is a favorable prognostic factor and may reflect the fact that most of the tumors in our study were less than 5 cm [24,25]. We also found no correlation between IRS grouping and prognosis, perhaps because most cases were of group II or III IRS type. Siddiqui et al. sourced 157 cases of pediatric sinonasal RMS (SNRMS) from the National Cancer Database and found using multivariate analyses that the IRS grouping was not a predictor for survival [26]. However, other analyses demonstrated an association between IRS group I and better prognoses and suggested that the IRS group is an independent prognostic factor for OS [26,27,28]. According to an analysis and treatment study by Darwish et al., major prognostic factors for survival in childhood RMS of the head and neck were embryonal histology, orbital site, extent of disease, and the use of surgery and radiation for orbital tumors [29].

Unlike earlier studies, we found no correlation between age at diagnosis and survival; the survival rate of our young cases (9 months to 10 years) was 96% and that of cases at older ages (≥10 years) was 93%. Infants under one year of age with orbital RMS reportedly have particularly poor prognosis, with 46% of them succumbing to the poorly understood aggressive nature of RMS in infancy [28,30]. The only infant under one year of age at presentation in our series showed persistent complete remission at long term follow-up.

Most tumors in our cohort (68%) were in the supranasal quadrant. We found no correlation between tumor orbital quadrant positioning and OS or EFS, but a significant association was found between tumor depth and EFS. Recurrence and exenteration as the final treatment were more likely in intraconal and apical lesions than extraconal or non-apical lesions. Most recurrence cases (80%) had intraconal/apical involvement with no influence on overall survival rate. Thus, among our study population we found out that intraconal and deep orbit involvement may be risk factors for recurrence and may reduce the chance of EFS.

In this study, we report long-term adverse events after local treatments for RMS, in which 33% of cases were diagnosed as having cataract (three underwent cataract surgery, and all cases had functional visual acuity 0.5 or better). We found that 5.1% of cases experienced mild to moderate dry eye, and 7.7% developed enophthalmos and bone hypoplasia. No enucleations were found in our cohort. Combined historic pooled data from the United States and Europe suggest that 51% to 82% of chemotherapy- and radiotherapy-treated children with RMS develop cataracts, 29% to 59% develop orbital hypoplasia, 54% to 70% experience reduced vision, and 11% to 14% may require enucleation for pain relief. According to a recent publication by Graef et al., the most common long-term complications were bony hypoplasia/facial asymmetry (40.3%) and keratopathy/dry eye (31.2%). Poor visual acuity (≤20/200) was noted in 13 (16.9%) patients, with 5 (6.5%) patients requiring an exenteration. In contrast, our cohort had lower adverse event rates than previously reported; these results can be explained by a substantial number of patients having received brachytherapy in our study [1,14,31,32].

The rate of event-free survival in our study was 63%, which is lower than described in previous publications [1]. Lower EFS was influenced by 9 PMN extension cases. It was also largely influenced by patient treated without radiotherapy (n = 10) according to the initial International Society of Pediatric Oncology approach, in which among the recurrence cases were five cases without any radiotherapy treatment. This conclusion is in accordance with Oberlin et al., showing that the SIOP-MMT approach results in lower EFS with equal OS [1]. In Yuan Wen et al.’s publication, radiation therapy as a component of initial treatment was shown to improve EFS in pediatric head and neck rhabdomyosarcoma patients by enhancing local control, and non-initial radiation therapy was identified as an independent risk factor for OS and EFS [33]. According to IRS group protocols, orbital RMS has a good prognosis, with preserved function of the eye. Primary chemotherapy followed by radiation therapy is the recommended treatment modality. In our updated treatment protocol, incisional biopsy was part of the primary diagnostic procedures before chemotherapy treatment initiation, with only two cases consisting of primary excisional biopsy. When the tumors were inaccessible for surgery and brachytherapy either due to apical location or extra ocular muscle involvement, EBRT or proton beam was indicated as primary treatment and exenteration was only adopted as final treatment approach combined with brachytherapy as a salvage treatment after failure of previous radiotherapy, either EBRT or AMORE [1,21,34].

An important limitation of this study was its retrospective design and the small sample size, impeding statistical analyses.

## 5. Conclusions

In summary, this study demonstrates the importance of achieving local tumor control with preservation of form and function in 77% of our study population treated with an eye-sparing treatment approach with a high (95%) survival rate.

## Figures and Tables

**Table 1:** Consecutive patients according to the intergroup rhabdomyosarcoma study classification.

Group	Description	NO. (%)
I	Completely resected localized disease implying both gross impression resection and microscopic confirmation of complete resection and absence of regional lymph node involvement	2 (5.1%)
Ia	Confined to muscle or organ of origin	0
II	Localized tumor, grossly removed with (a) microscopically involved margins, (b) involved grossly resected regional lymph nodes, or (c) both	11 (28.2%)
III	Incomplete resection with biopsy or gross residual disease at site of origin or in regional lymph nodes	20 (51.3%)
IV	Distant metastases present at onset	0
No information	No information	6 (15.4)

**Table 2:** RMS pretreatment staging based on TNM classification *.

Stage I	T1, Tumor localized in the organ or tissue of origin N0, No evidence of regional lymph node involvement M0, No evidence of distant metastasis
Stage II	T2, Tumor involving one or more contiguous organs or tissues or with adjacent malignant effusion N0 M0
Stage III	Any T N 1, Evidence of regional lymph node involvement M0
Stage IV	Any T Any N M1, Evidence of distant metastases

* Tx—primary tumor cannot be assessed; T0—no evidence of primary tumor; T1—tumor 15 mm or less in greatest dimension; T2—tumor more than 15 mm in greatest dimension without invasion of globe or bony wall; T3—tumor of any size with invasion of orbital tissue or bony walls; T4—tumor invasion of globe or periorbital structure, such as eyelids, temporal fossa, nasal cavity and paranasal sinuses or central nervous system. Nx—regional lymph nodes cannot be assessed; N0—no regional lymph node metastasis; N1—regional lymph node metastasis. M0—no distance metastasis; M1—distance metastasis.

**Table 3:** Recurrent/residual cases description.

Patients Serial No.	Primary Treatment	Secondary Treatment	Tertiary Treatment	Final Outcome	Histology
3	AMORE	iBio + EBT + chem	Ex + chemo + brach	CR	Embryonal
12	AMORE	AMORE	Ex + chemo + brach	CR	Embryonal
17	AMORE	Ex + chemo + brach	-	CR	Embryonal
33	AMORE	iBio + Proton + chemo	Ex + chemo + brach	CR	Embryonal
27	AMORE	AMORE	Ex + chemo + brach	death	Embryonal
22	AMORE	AMORE	Lack of information	death	Embryonal
6	iBio + chemo	Exen + chemo + brach	-	CR	Alveolar
19	iBio + chemo	AMORE	-	CR	Embryonal
20	iBio + chemo + EBT	AMORE	Ex + chemo + brach	CR	Embryonal
40	iBio + chemo	AMORE	-	CR	Embryonal
41	iBio + Chemo + proton	Ex + chemo + brach		CR	Embryonal
42	iBio + Chemo + proton	iBio + chemo + EBT	AMORE	CR	Embryonal
7	iBio + chemo	AMORE	Ex + chemo + brach	CR	Embryonal
10	iBio + chemo	iBio + chemo + EBT	Ex + chemo + brach	CR	Embryonal
					Embryonal

Keywords: eBio—excisional biopsy; iBio—incisional biopsy; Chemo—chemotherapy; EBT—external beam therapy; Stereo—stereotactic radiotherapy; CR—complete remission; Ex—exenteration.

**Table 4:** Local and systemic tumor outcomes in 31 patients of primary RMS.

Outcome	NO. (%)
Local tumor outcome (more than 5 years follow up) Regression Residual enhancement Recurrence Persistent **	Total * 31 (100) 29 (93.33) 27 (87) 15 (48) 2 (6.5)
Regional outcome No lymph node spread Lymph node spread	31 (100) 0
Systemic outcome No distant metastasis Distant metastasis ^¥^	30 1
Final status Alive Dead	29(93.6) 2 (6.4)

* Exclusion of 8 cases with follow-up less than 5 years. ¥ Full metastasis recovery was achieved after primary treatment of iBio + chemotherapy + EBT. ** Two cases with residual persistence after they had cured exenteration. Two different cases of death have been described in recurrence cases.

## Data Availability

The original contributions presented in this study are included in the article. Further inquiries can be directed to the corresponding author.
